# Supplementary material for: Fish species richness is associated with the availability of landscape components across seasons in the Amazonian floodplain
Source: PeerJ. 2018 Jun 21;6:e5080. doi: 10.7717/peerj.5080 (PMC6015757; doi:10.7717/peerj.5080)
Supplement: Supplemental Information 1 [file peerj-06-5080-s001.docx]

S1. Studied lakes: their geographical coordinates, number of samplings in each phase of the hydrological cycle (HW = high water, LW = low water), sampling effort (in hours of the net in the water) and area (km^2^).

| Lake | Latitude | Longitude | Samplings Number | | Sampling Effort (hours) | | Lake’s area (km^2)^ | |
| --- | --- | --- | --- | --- | --- | --- | --- | --- |
|  |  |  | HW | LW | HW | LW | HW | LW |
| Ananá | 03°54'25.0"S | 61°40'34.0"W | 8 | 8 | 192 | 192 | 3.595 | 1.028 |
| Araçá | 03°45'54.5"S | 62°20'41.9"W | 8 | 8 | 192 | 192 | 1.572 | 0.646 |
| Baixio | 03°17'28.0"S | 60°04'43.7"W | 8 | 8 | 192 | 192 | 3.833 | 0.697 |
| Cacauzinho | 03°40'07.0"S | 69°52'16.3"W | 1 | 1 | 48 | 48 | 0.145 | 0.075 |
| Calado | 03°18'50.0"S | 60°34'51.0"W | 3 | 2 | 144 | 96 | NB | 0.255 |
| Camaleão | 03°39'51.6"S | 60°54'35.3"W | 1 | 1 | 48 | 48 | 0.246 | 0.145 |
| Camboa | 03°35'03.2"S | 69°59'38.5"W | 1 | 1 | 48 | 48 | NB | 0.263 |
| Central | 03°15'13.8"S | 59°58'12.3"W | 3 | 2 | 144 | 96 | NB | 0.340 |
| Iauara | 03°36'29.2"S | 61°16'45.9"W | 8 | 8 | 192 | 192 | NB | 0.144 |
| Maracá | 03°50'40.6"S | 62°34'30.1"W | 8 | 8 | 192 | 192 | 0.365 | 0.331 |
| Padre | 03°11'47.8"S | 59°55'53.3"W | 3 | 2 | 144 | 96 | NB |  |
| Poraqué | 03°57'29.6"S | 63°09'48.2"W | 8 | 8 | 192 | 192 | 0.136 | 0.085 |
| Preto | 03°21'04.2"S | 60°37'23.5”W | 8 | 8 | 192 | 192 | 1.870 | 1.572 |
| Sacambú | 03°98’12.0”S | 59°56’26.0"W | 3 | 2 | 144 | 96 | NB |  |
| Santo Antonio | 03°14'42.9"S | 60°14'40.9"W | 3 | 2 | 144 | 96 | NB |  |
