# Supplementary material for: Fish species richness is associated with the availability of landscape components across seasons in the Amazonian floodplain
Source: PeerJ. 2018 Jun 21;6:e5080. doi: 10.7717/peerj.5080 (PMC6015757; doi:10.7717/peerj.5080)
Supplement: Supplemental Information 2 — Total S = total species richness, Carnivorous S = carnivorous species richness, Omnivorous S = omnivorous species richness, Herbivorous S = herbivorous species richness, Migrant S = migrant species richness, Resident S = resident species richness, Pelagic P = pelagic species richness, Benthopelagic B = benthopelagic species richness, OW = open water, FH = flooded herbaceous, FS = flooded shrubs, FF = flooded forest, n = number of collected fish. [file peerj-06-5080-s002.docx]

S2. Summary of assumptions tested by each model, where: Total S = total species richness, Carnivorous S = carnivorous species richness, Omnivorous S = omnivorous species richness, Herbivorous S = herbivorous species richness, Migrant S = migrant species richness, Resident S = resident species richness, Pelagic P = pelagic species richness, Benthopelagic B = benthopelagic species richness, OW = open water, FH = flooded herbaceous, FS = flooded shrubs, FF = flooded forest, n = number of collected fish.

| Season/Buffer | Diversity level | Moran I | Variance Inflation Factor: Initial Model | Variance Inflation Factor: Final Model |
| --- | --- | --- | --- | --- |
| High water 500 | Total S  Carnivorous S  Omnivorous S  Herbivorous S  Migrants S  Residents S  Pelagics P  Benthopelagic B | P = 0.43  P = 0.21  P = 0.18  P = 0.54  P = 0.19  P = 0.54  P = 0.52  P = 0.48 | OW = 3.62, FH = 2.15, FS = 1.78  FF = 5.44, n = 1.42  OW = 3.56, FH = 2.21, FS = 1.79  FF = 5.59, n = 1.45  OW = 3.55, FH = 2.11, FS = 1.85  FF = 5.39, n = 1.45  OW = 4.10, FH = 2.19, FS = 1.86  FF = 5.77, n = 1.41  OW = 3.85, FH = 2.27, FS = 1.82  FF = 5.75, n = 1.41  OW = 3.45, FH = 2.06, FS = 1.74  FF = 5.19, n = 1.43  OW = 3.31, FH = 2.09, FS = 1.69  FF = 5.14, n = 1.38  OW = 3.83, FH =2.19, FS = 1.84  FF = 5.66, n= 1.43 | OW = 1.05, FH = 1.36,  FS = 1.32, n = 1.13  OW = 1.04, FH = 1.41,  FS = 1.35, n = 1.13  OW = 1.05, FH = 1.35,  FS = 1.31, n = 1.12  OW = 1.07, FH = 1.33,  FS = 1.31, n = 1.15  OW = 1.05, FH = 1.39,  FS = 1.34, n = 1.14  OW = 1.05, FH = 1.34,  FS = 1.30, n = 1.13  OW = 1.05, FH = 1.35,  FS = 1.30, n = 1.12  OW = 1.05, FH = 1.37  FS = 1.33, n = 1.14 |
| High water 1000 | Total S  Carnivorous S  Omnivorous S  Herbivorous S  Migrants S  Residents S  Pelagics P  Benthopelagic B | P = 0.26  P = 0.10  P = 0.10  P = 0.98  P = 0.09  P = 0.75  P = 0.23  P = 0.02 | OW = 5.02, FH = 2.23, FS = 1.45, FF = 6.21, n = 1.48  OW = 4.94, FH = 2.18, FS = 1.45  FF = 6.26, n = 1.53  OW = 4.69, FH = 2.16, FS = 1.46  FF = 5.87, n = 1.47  OW = 5.43, FH = 2.30, FS = 1.46  FF = 6.34, n = 1.47  OW = 5.26, FH = 2.36, FS = 1.44  FF = 6.53, n = 1.47  OW = 4.91, FH = 2.14, FS = 1.46  FF = 5.99, n = 1.49  OW = 4.67, FH = 2.17, FS = 1.44  FF = 6.12, n = 1.48  OW = 5.25, FH = 2.28, FS = 1.46  FF = 6.31, n = 1.48 | OW = 1.09, FH = 1.05,  FS = 1.20, n = 1.12  OW = 1.09, FH = 1.05,  FS = 1.19, n = 1.11  OW = 1.10, FH = 1.05,  FS = 1.18, n = 1.11  OW = 1.12, FH = 1.05,  FS = 1.23, n = 1.14  OW = 1.09, FH = 1.05,  FS = 1.21, n = 1.13  OW = 1.10, FH = 1.05,  FS = 1.20, n = 1.12  OW = 1.07, FH = 1.05  FS = 1.19, n = 1.13  OW = 1.11, FH = 1.05  FS = 1.21, n = 1.12 |
| High water 5000 | Total S  Carnivorous S  Omnivorous S  Herbivorous S  Migrants M  Residents R  Pelagics P  Benthopelagic B | P = 0.96  P = 0.27  P = 0.62  P = 0.14  P = 0.23  P = 0.21  P = 0.82  P = 0.56 | OW = 1.97, FH = 1.27, FS =2.42,  FF = 3.47, n = 1.48  OW = 1.97, FH =1.33, FS = 2.58, FF = 3.45, n = 1.45  0W = 2.04, FH = 1.31, FS = 2.37, FF = 3.44, n = 1.46  OW = 1.97, FH = 1.26, FS = 2.32, FF = 3.45, n = 1.47  OW = 1.99, FH = 1.26, FS = 2.43,  FF = 3.50, n = 1.46  OW = 1.96, FH = 1.28, FS = 2.42,  FF = 3.45, n = 1.47  OW = 1.91, FH = 1.23, FS = 1.51,  FF = 1.98, n = 1.49  OW = 2.02, FH = 1.30, FS = 2.39,  FF = 3.44, n = 1.45 | OW = 1.24, FH = 1.17,  FS = 1.64, n = 1.30  OW = 1.30, FH = 1.21,  FS = 1.69, n = 1.30  OW = 1.24, FH = 1.18,  FS = 1.63, n = 1.28  OW = 1.21, FH = 1.16,  FS = 2.32, n = 1.47  OW = 1.25, FH = 1.16,  FS = 1.64, n = 1.30  OW = 1.24, FH = 1.17,  FS = 1.64, n = 1.29  OW = 1.91, FH = 1.23,  FS = 1.51, FF = 1.98,  n = 1.49  OW = 1.25, FH = 1.18,  FS = 1.63, n = 1.28 |
| Low water 500 | Total S  Carnivorous S  Omnivorous S  Herbivorous S  Migrants M  Residents R  Pelagics P  Benthopelagic B | P = 0.11  P = 0.19  P = 0.06  P = 0.97  P = 0.47  P = 0.85  P = 0.74  P = 0.47 | OW = 1.48, FH = 1.25,  FF = 1.52, n = 1.04  OW =1.40, FH = 1.17,  FF = 1.45, n = 1.06  OW = 1.43, FH = 1.20,  FF = 1.48, n = 1.06  OW = 1.44, FH = 1.21,  FF = 1.50, n = 1.04  OW = 1.44, FH = 1.23,  FF = 1.51, n = 1.05  OW = 1.51, FH = 1.25,  FF = 1.53, n = 1.05  OW = 1.45, FH = 1.22,  FF = 1.51, n = 1.05  OW = 1.49, FH = 1.26,  FF = 1.53, n = 1.05 | OW = 1.48, FH = 1.25,  FF = 1.52, n = 1.04  OW = 1.40, FH = 1.17,  FF = 1.45, n = 1.06  OW = 1.43, FH = 1.20,  FF = 1.48, n = 1.06  OW = 1.44, FH = 1.21,  FF = 1.50, n = 1.04  OW = 1.44, FH = 1.23,  FF = 1.51, n = 1.05  OW = 1.51, FH = 1.25,  FF = 1.53, n = 1.05  OW = 1.45, FH = 1.22,  FF = 1.51, n = 1.05  OW = 1.49, FH = 1.26,  FF = 1.53, n = 1.05 |
| Low water 1000 | Total S  Carnivorous S  Omnivorous S  Herbivorous S  Migrants M  Residents R  Pelagics P  Benthopelagic B | P = 0.67  P = 0.03*  P = 0.02*  P = 0.85  P = 0.68  P = 0.23  P = 0.95  P = 0.70 | OW = 2.97, FH = 2.14,  FF = 3.71, n = 1.22  OW = 2.81, FH = 2.01,  FF = 3.58, n = 1.19  OW = 2.79, FH = 2.05,  FF = 3.59, n = 1.19  OW = 2.91, FH = 2.02,  FF = 3.65, n = 1.01  OW = 2.81, FH = 2.15,  FF = 3.66, n = 1.22  OW = 3.09, FH = 2.10,  FF = 3.73, n = 1.22  OW = 3.01, FH = 2.18,  FF = 3.75, n = 1.22  OW = 2.95, FH =2.11,  FF = 3.67, n = 1.22 | OW = 1.07, FH = 1.06,  n = 1.02  OW = 1.05, FH = 1.03,  n = 1.01  OW = 1.05, FH = 1.04,  n = 1.01  OW = 1.06, FH = 1.04,  n = 1.01  OW = 1.06, FH = 1.04,  n = 1.01  OW = 1.08, FH = 1.06,  n = 1.02  OW = 1.07, FH = 1.05,  N = 1.01  OW = 1.07, FH = 1.05,  N = 1.02 |
| Low water 5000 | Total S  Carnivorous S  Omnivorous S  Herbivorous S  Migrants M  Residents R  Pelagics P  Benthopelagic B | P = 0.98  P = 0.38  P = 0.53  P = 0.13  P = 0.34  P = 0.26  P = 0.35  P = 0.98 | OW = 2.18, FH = 1.52, FS = 1.54,  FF = 2.29, n = 1.22  OW = 2.06, FH = 1.59, FS = 1.52,  FF = 2.31, n = 1.21  OW = 1.95, FH = 1.51, FS = 1.53,  FF = 2.06, n = 1.20  OW = 2.02, FH = 1.52, FS = 1.52,  FF = 2.12, n = 1.19  OW = 2.28, FH = 1.62, FS = 1.54,  FF = 2.53, n = 1.22  OW = 2.08, FH = 1.46, FS = 1.52,  FF = 2.13, n = 1.21  OW = 2.28, FH = 1.59, FS = 1.56,  FF = 2.43, n = 1.21  OW = 2.12, FH = 1.49, FS =1.52,  FF = 2.24, n = 1.22 | OW = 1.50, FH, 1.17,  FS = 1.48, n = 1.10  OW = 1.48, FH, 1.19,  FS = 1.49, n = 1.08  OW = 1.48, FH = 1.19,  FS = 1.50, n = 1.09  OW = 1.49, FH = 1.17,  FS = 1.49, n = 1.09  OW = 1.50, FH = 1.17,  FS = 1.49 n = 1.10  OW = 1.49, FH = 1.15,  FS = 1.47, n = 1.10  OW = 1.51, FH = 1.18,  FS = 1.49, n = 1.10  OW = 1.48, FH = 1.16,  FS = 1.47, n = 1.22 |
