# Supplementary material for: Fish species richness is associated with the availability of landscape components across seasons in the Amazonian floodplain
Source: PeerJ. 2018 Jun 21;6:e5080. doi: 10.7717/peerj.5080 (PMC6015757; doi:10.7717/peerj.5080)
Supplement: Supplemental Information 3 — Where x = present and … = absent. And type of functional group as C = carnivorous, I = omnivororus, H = herbivorous, R = resident, M = migratory, P = pelagic and B = benthopelagic. [file peerj-06-5080-s003.docx]

S3. List of species by lake and season of the hydrological cycle (H = high water and L = low water). Where x = present and … = absent. And type of functional group as C = carnivorous, I = omnivororus, H = herbivorous, R = resident, M = migratory, P = pelagic and B = benthopelagic.

|  |  | **Ananá** | | **Araça** | | **Baixio** | | **Cacauzinho** | | **Calado** | | **Camaleão** | | **Camboa** | | **Central** | | **Iauara** | | **Maracá** | | **Padre** | | **Poraqué** | | **Preto** | | **Sacambú** | | **St. Antônio** | |
| --- | --- | --- | --- | --- | --- | --- | --- | --- | --- | --- | --- | --- | --- | --- | --- | --- | --- | --- | --- | --- | --- | --- | --- | --- | --- | --- | --- | --- | --- | --- | --- |
|  |  | **H** | **L** | **H** | **L** | **H** | **L** | **H** | **L** | **H** | **L** | **H** | **L** | **H** | **L** | **H** | **L** | **H** | **L** | **H** | **L** | **H** | **L** | **H** | **L** | **H** | **L** | **H** | **L** | **H** | **L** |
| **LEPIDOSIRENIFORMES** | Type |  |  |  |  |  |  |  |  |  |  |  |  |  |  |  |  |  |  |  |  |  |  |  |  |  |  |  |  |  |  |
| **Lepidosirenidae** |  |  |  |  |  |  |  |  |  |  |  |  |  |  |  |  |  |  |  |  |  |  |  |  |  |  |  |  |  |  |  |
| *Lepidosideren paradoxa* | H, R | -- | -- | -- | -- | -- | -- | -- | -- | -- | -- | -- | X | -- | -- | -- | -- | -- | -- | -- | -- | -- | -- | -- | -- | -- | -- | -- | -- | -- | -- |
| **OSTEOGLOSSIFORMES** |  |  |  |  |  |  |  |  |  |  |  |  |  |  |  |  |  |  |  |  |  |  |  |  |  |  |  |  |  |  |  |
| **Osteoglossidae** |  |  |  |  |  |  |  |  |  |  |  |  |  |  |  |  |  |  |  |  |  |  |  |  |  |  |  |  |  |  |  |
| *Osteoglossum bicirrhosum* | C, R, B | X | X | X | X | -- | X | X | X | -- | X | X | -- | X | X | -- | X | X | X | X | X | X | -- | -- | -- | X | X | -- | X | -- | -- |
| **CLUPEIFORMES** |  |  |  |  |  |  |  |  |  |  |  |  |  |  |  |  |  |  |  |  |  |  |  |  |  |  |  |  |  |  |  |
| **Engraulidae** |  |  |  |  |  |  |  |  |  |  |  |  |  |  |  |  |  |  |  |  |  |  |  |  |  |  |  |  |  |  |  |
| *Anchoviella jamesi* | R | -- | -- | -- | -- | -- | -- | -- | -- | -- | -- | -- | -- | -- | -- | -- | -- | -- | -- | -- | -- | -- | -- | -- | -- | -- | -- | -- | -- | X | -- |
| *Jurengraulis juruensis* | R | -- | -- | -- | -- | -- | -- | -- | -- | -- | -- | -- | -- | -- | -- | -- | -- | -- | -- | -- | -- | -- | X | -- | -- | -- | -- | -- | -- | -- | -- |
| *Lycengraulius batesii* | C, R | -- | X | -- | X | X | -- | -- | -- | X | X | -- | -- | -- | -- | -- | X | -- | -- | X | -- | -- | X | X | -- | -- | -- | -- | X | -- | X |
| **Pristigasteridae** |  |  |  |  |  |  |  |  |  |  |  |  |  |  |  |  |  |  |  |  |  |  |  |  |  |  |  |  |  |  |  |
| *Ilisha amazonica* | R | -- | -- | -- | -- | -- | -- | -- | -- | -- | X | -- | -- | -- | -- | -- | -- | -- | -- | -- | -- | -- | X | X | -- | -- | -- | -- | X | -- | X |
| *Pellona castelnaeana* | C, M, P | X | X | X | X | X | X | X | X | X | X | X | X | X | X | X | X | X | X | X | X | -- | X | -- | -- | -- | -- | -- | X | X | X |
| *Pellona flavipinnis* | C, M, P | X | X | X | X | X | X | -- | X | -- | X | X | X | X | X | X | X | -- | X | X | X | -- | X | -- | -- | -- | -- | X | X | X | X |
| *Pristigaster whiteheadi* | R | -- | -- | -- | -- | -- | -- | -- | -- | -- | -- | -- | -- | -- | -- | X | -- | -- | -- | -- | -- | -- | X | -- | -- | -- | -- | -- | -- | -- | X |
| **CHARACIFORMES** |  |  |  |  |  |  |  |  |  |  |  |  |  |  |  |  |  |  |  |  |  |  |  |  |  |  |  |  |  |  |  |
| **Curimatidae** |  |  |  |  |  |  |  |  |  |  |  |  |  |  |  |  |  |  |  |  |  |  |  |  |  |  |  |  |  |  |  |
| *Curimata inornata* | M, B | X | X | X | X | X | X | -- | -- | -- | X | -- | X | -- | X | -- | -- | -- | -- | X | X | -- | X | X | X | X | X | X | X | -- | -- |
| *Curimata knerii* | M | -- | -- | -- | -- | -- | -- | -- | -- | -- | -- | X | -- | -- | -- | -- | -- | -- | -- | -- | -- | -- | -- | -- | -- | -- | -- | -- | -- | -- | -- |
| *Curimata ocellata* | R, B | -- | -- | -- | -- | X | -- | -- | -- | X | -- | -- | -- | -- | -- | -- | X | -- | -- | X | -- | -- | -- | -- | -- | -- | -- | -- | -- | -- | -- |
| *Curimata vittata* | R, B | -- | X | -- | X | -- | -- | -- | -- | -- | -- | -- | -- | X | -- | -- | -- | -- | X | -- | X | X | -- | -- | -- | -- | -- | -- | X | -- | -- |
| *Curimatella meyeri* | R | X | X | -- | X | -- | X | X | X | -- | -- | X | X | X | X | X | X | X | X | X | X | X | -- | X | X | X | X | X | X | X | X |
| *Cyphocharax abramoides* | R | -- | -- | -- | -- | -- | -- | -- | -- | -- | -- | -- | -- | -- | -- | -- | -- | -- | -- | X | -- | -- | -- | -- | -- | -- | -- | -- | -- | -- | -- |
| *Potamorhina altamazonica* | M, B | X | X | X | X | X | X | X | X | -- | X | X | X | X | X | X | X | X | X | X | X | X | X | X | X | X | X | X | X | X | X |
| *Potamorhina latior* | M | X | X | X | X | X | X | X | X | -- | X | X | X | X | X | X | X | X | X | X | X | X | X | X | X | X | X | X | X | X | X |
| *Potamorhina pristigaster* | R, B | X | X | X | X | -- | -- | X | X | -- | -- | X | X | X | X | X | -- | X | X | X | X | -- | -- | X | X | X | X | -- | -- | -- | -- |
| *Psectrogaster amazonica* | M, B | X | X | X | X | X | X | X | X | -- | X | X | X | X | X | -- | -- | X | X | X | X | X | -- | X | X | X | X | X | X | X | X |
| *Psectrogaster essequibensis* | M | -- | -- | -- | -- | -- | -- | X | -- | -- | -- | -- | -- | -- | -- | -- | -- | X | X | -- | -- | -- | -- | X | X | -- | -- | -- | -- | -- | -- |
| *Psectrogaster rutiloides* | M, B | X | X | X | X | X | X | X | X | -- | -- | X | X | X | X | X | X | X | X | X | X | X | X | X | X | X | X | X | X | X | X |
| **Prochilodontidae** |  |  |  |  |  |  |  |  |  |  |  |  |  |  |  |  |  |  |  |  |  |  |  |  |  |  |  |  |  |  |  |
| *Prochilodus nigricans* | M, B | X | X | X | X | X | X | X | X | X | -- | X | -- | X | X | X | X | X | X | X | X | X | X | X | X | X | X | X | -- | X | X |
| *Semaprochilodus insignis* | M, B | X | X | X | X | X | X | X | X | X | X | X | X | X | X | X | -- | X | X | X | X | X | X | X | X | X | X | X | X | X | X |
| *Semaprochilodus taeniurus* | M, B | X | X | X | X | X | X | X | X | X | -- | X | X | X | X | X | X | -- | X | X | X | X | X | -- | X | -- | -- | -- | X | -- | X |
| **Hemiodontidae** |  |  |  |  |  |  |  |  |  |  |  |  |  |  |  |  |  |  |  |  |  |  |  |  |  |  |  |  |  |  |  |
| *Anodus elongatus* | M | X | X | X | X | X | X | X | X | X | X | X | X | X | X | X | X | X | X | X | X | X | X | X | -- | -- | -- | X | -- | X | X |
| *Anodus orinocensis* | M | -- | -- | -- | -- | -- | -- | -- | -- | X | X | -- | X | -- | -- | -- | -- | -- | -- | -- | -- | -- | -- | -- | -- | -- | -- | -- | -- | X | X |
| *Hemiodus atranalis* | R | -- | -- | -- | -- | -- | -- | -- | -- | X | -- | -- | -- | -- | -- | -- | -- | -- | -- | -- | -- | -- | -- | X | -- | -- | -- | -- | -- | -- | -- |
| *Hemiodus immaculatus* | O, M, B | X | -- | -- | X | -- | X | X | X | X | X | X | -- | X | X | -- | -- | -- | X | X | X | -- | -- | X | X | X | -- | X | -- | X | X |
| *Hemiodus microlepis* | O, M | X | -- | -- | X | -- | X | -- | -- | -- | -- | X | -- | -- | -- | -- | X | -- | -- | -- | X | -- | -- | X | -- | -- | -- | -- | -- | -- | X |
| *Hemiodus unimaculatus* | H, M, B | -- | -- | X | -- | -- | X | -- | -- | X | -- | X | -- | -- | -- | -- | X | -- | X | X | X | -- | X | X | X | -- | -- | X | X | -- | X |
| *Hemiodus* sp. | O, P | X | X | X | X | X | X | X | X | -- | X | X | X | X | X | X | X | -- | X | X | X | -- | X | X | -- | X | -- | X | X | X | X |
| **Anostomidae** |  |  |  |  |  |  |  |  |  |  |  |  |  |  |  |  |  |  |  |  |  |  |  |  |  |  |  |  |  |  |  |
| *Anostomoides laticeps* | M | -- | -- | -- | -- | -- | X | -- | -- | -- | -- | -- | -- | -- | -- | -- | -- | -- | -- | -- | -- | -- | -- | -- | -- | -- | X | -- | -- | -- | -- |
| *Laemolyta proxima* | M | -- | -- | -- | -- | -- | -- | -- | -- | X | -- | X | -- | X | -- | -- | -- | -- | -- | -- | -- | -- | -- | -- | -- | -- | -- | -- | -- | -- | -- |
| *Laemolyta taeniata* | M | -- | -- | -- | -- | -- | -- | -- | -- | -- | X | -- | -- | -- | -- | -- | -- | -- | -- | -- | -- | -- | -- | -- | -- | -- | -- | -- | -- | -- | -- |
| *Leporinus amazonicus* | M | -- | -- | -- | -- | -- | -- | -- | -- | X | -- | -- | -- | X | -- | -- | -- | -- | -- | -- | -- | -- | -- | -- | -- | -- | -- | -- | -- | -- | -- |
| *Leporinus friderici* | H, R, B | X | X | X | X | -- | X | X | X | X | X | X | X | X | X | X | X | X | X | X | X | X | -- | X | X | X | X | X | X | -- | X |
| *Leporinus trifasciatus* | O, M, B | X | X | X | X | X | X | X | X | X | X | X | X | X | X | X | X | X | X | X | X | X | X | X | X | X | -- | -- | X | X | -- |
| *Leporinus* spp. |  | -- | -- | X | X | X | -- | -- | -- | -- | -- | -- | -- | -- | -- | -- | -- | -- | -- | X | -- | -- | -- | -- | -- | -- | -- | -- | -- | -- | -- |
| *Rhytiodus argenteofuscus* | O, M | X | X | X | X | X | X | X | -- | X | X | X | -- | X | -- | -- | -- | X | X | X | -- | X | -- | -- | X | -- | -- | -- | -- | -- | -- |
| *Rhytiodus microlepis* | H, M, B | X | X | X | X | X | X | X | X | -- | -- | X | X | X | X | -- | X | X | X | X | -- | X | X | X | X | X | -- | X | X | -- | X |
| *Schyzodon fasciatus* | H, M, B | X | X | X | X | X | X | X | X | X | -- | X | X | X | X | X | X | X | X | X | X | X | X | -- | X | X | X | X | X | X | X |
| *Schyzodon vittatus* | H, M, B | X | -- | -- | X | X | X | -- | -- | X | -- | -- | -- | -- | -- | X | -- | -- | X | X | X | -- | -- | X | X | -- | X | X | X | X | X |
| **Characidae** |  |  |  |  |  |  |  |  |  |  |  |  |  |  |  |  |  |  |  |  |  |  |  |  |  |  |  |  |  |  |  |
| *Chalceus erythrurus* | R | X | X | X | X | -- | X | X | X | -- | -- | X | X | -- | -- | -- | X | -- | -- | X | X | -- | X | X | -- | X | X | X | X | -- | -- |
| *Ctenobrycon hauxwellianus* | R | X | -- | -- | -- | -- | -- | X | -- | -- | -- | -- | -- | X | -- | -- | -- | -- | -- | -- | -- | -- | -- | -- | -- | X | X | -- | -- | -- | -- |
| *Tetragonopterus argenteus* | C, R | -- | -- | -- | X | -- | -- | X | -- | -- | -- | X | -- | X | -- | -- | -- | -- | -- | -- | -- | -- | X | -- | -- | -- | -- | -- | -- | -- | -- |
| *Tetragonopterus chalceus* | R | -- | -- | X | X | X | -- | -- | X | -- | -- | -- | -- | -- | -- | -- | -- | -- | -- | X | X | -- | X | -- | -- | -- | -- | -- | -- | -- | -- |
| **Bryconidae** |  |  |  |  |  |  |  |  |  |  |  |  |  |  |  |  |  |  |  |  |  |  |  |  |  |  |  |  |  |  |  |
| *Brycon amazonicus* | O, M, B | X | X | X | X | X | X | X | X | -- | -- | X | -- | X | X | -- | X | X | X | X | X | X | X | X | X | X | X | X | X | X | X |
| *Brycon melanopterus* | O, M | X | X | X | X | -- | X | -- | X | X | X | X | -- | X | X | -- | X | X | -- | X | X | X | X | X | -- | X | -- | -- | -- | -- | X |
| **Iguanodectidae** |  |  |  |  |  |  |  |  |  |  |  |  |  |  |  |  |  |  |  |  |  |  |  |  |  |  |  |  |  |  |  |
| *Bryconops caudomaculatus* | R | -- | -- | -- | -- | -- | -- | -- | -- | -- | X | -- | -- | -- | -- | -- | -- | -- | -- | -- | -- | -- | -- | -- | X | -- | -- | -- | -- | -- | X |
| **Serrasalmidae** |  |  |  |  |  |  |  |  |  |  |  |  |  |  |  |  |  |  |  |  |  |  |  |  |  |  |  |  |  |  |  |
| *Catoprion mento* | C, R, B | X | X | -- | -- | -- | -- | -- | -- | -- | X | -- | -- | -- | -- | -- | -- | -- | -- | X | -- | -- | -- | -- | -- | -- | -- | -- | -- | -- | -- |
| *Colossoma macropomum* | O, M, P | X | X | X | X | X | X | X | -- | -- | -- | X | -- | X | X | X | X | X | X | X | X | X | X | X | -- | X | X | X | X | -- | -- |
| *Metynnis argenteus* | R, P | -- | -- | -- | -- | X | X | -- | -- | -- | -- | -- | -- | -- | -- | -- | -- | X | -- | X | -- | -- | -- | X | -- | X | X | -- | -- | -- | -- |
| *Metynnis hypsauchen* | H, R, P | X | X | X | -- | X | -- | -- | -- | X | X | -- | -- | -- | -- | X | X | -- | -- | X | -- | -- | -- | -- | -- | -- | -- | X | X | -- | -- |
| *Moenkhausia lepidura* | R | -- | -- | -- | -- | -- | -- | -- | -- | -- | -- | -- | -- | -- | -- | -- | -- | -- | -- | -- | -- | -- | -- | -- | X | -- | X | -- | -- | -- | -- |
| *Myleus torquatus* | R | X | -- | -- | -- | X | -- | -- | -- | -- | -- | -- | -- | -- | -- | -- | -- | -- | -- | -- | -- | -- | -- | -- | -- | -- | -- | -- | -- | -- | -- |
| *Myloplus rubripinnis* | R | -- | -- | -- | -- | -- | -- | -- | -- | -- | -- | X | -- | -- | -- | -- | -- | X | -- | -- | -- | -- | -- | -- | -- | X | -- | -- | -- | -- | -- |
| *Mylossoma aureum* | M, B | X | X | X | X | X | X | X | X | X | X | X | X | X | X | X | X | X | X | X | X | -- | X | -- | -- | -- | -- | X | X | X | X |
| *Mylossoma duriventre* | O, M, P | X | X | X | X | X | X | X | X | X | X | X | X | X | X | X | X | X | X | X | X | -- | X | X | X | X | X | X | X | X | X |
| *Piaractus brachypomus* | O, M, P | X | X | X | X | -- | X | -- | -- | X | X | X | -- | X | X | -- | -- | X | X | X | X | -- | -- | X | X | X | X | -- | X | -- | -- |
| *Pristobrycon calmoni* | O, R, B | X | X | X | X | X | X | -- | -- | -- | -- | -- | -- | -- | -- | -- | X | X | X | X | -- | -- | X | -- | -- | X | -- | -- | -- | -- | -- |
| *Pygocentrus nattereri* | C, R, P | X | X | X | X | X | X | X | X | X | -- | X | X | X | X | X | X | X | X | X | X | X | X | X | X | X | X | X | X | X | X |
| *Roeboides myersii* | R, B | -- | X | -- | X | -- | X | -- | X | -- | -- | X | X | X | X | -- | -- | -- | X | X | -- | X | X | -- | -- | -- | -- | -- | X | -- | X |
| *Serrasalmus altispinis* | C, R, P | X | X | X | X | X | X | -- | -- | -- | -- | -- | -- | -- | -- | -- | -- | X | X | X | X | -- | -- | -- | -- | X | X | -- | -- | -- | -- |
| *Serrasalmus eigenmanni* | R, B | X | X | -- | -- | -- | X | -- | -- | -- | -- | -- | -- | -- | -- | -- | -- | -- | X | X | -- | -- | -- | -- | -- | X | -- | -- | -- | -- | -- |
| *Serrasalmus elongatus* | C, R, B | X | X | X | X | X | X | X | X | -- | -- | X | X | X | X | -- | X | X | X | X | X | X | X | X | X | X | X | X | X | X | X |
| *Serrasalmus rhombeus* | C, R, B | X | X | X | X | X | X | X | X | -- | X | X | X | X | X | -- | X | X | X | X | X | X | X | X | X | -- | X | X | X | X | X |
| *Serrasalmus serrulatus* | R, B | -- | X | -- | X | -- | X | X | X | -- | -- | X | -- | X | X | X | X | -- | X | X | -- | X | X | -- | -- | -- | -- | -- | -- | -- | -- |
| *Serrasalmus spilopleura* | O, R, B | X | X | X | X | X | X | X | X | X | X | X | X | X | X | X | X | X | X | X | X | -- | X | -- | X | X | X | X | X | X | X |
| **Triportheidae** |  |  |  |  |  |  |  |  |  |  |  |  |  |  |  |  |  |  |  |  |  |  |  |  |  |  |  |  |  |  |  |
| *Agoniates anchovia* | M | -- | -- | -- | -- | -- | -- | -- | -- | -- | -- | -- | -- | -- | -- | -- | -- | -- | -- | -- | -- | -- | X | -- | -- | -- | -- | -- | X | -- | -- |
| *Triportheus albus* | O, M, P | X | X | X | X | X | X | X | X | X | X | X | X | X | X | X | X | X | X | X | X | X | X | X | X | X | X | X | X | X | X |
| *Triportheus angulatus* | O, M, P | X | X | X | X | X | X | X | X | X | X | X | X | X | X | X | X | X | X | X | X | X | X | X | X | X | X | X | X | X | X |
| *Triportheus auritus* | M | X | X | -- | X | X | -- | -- | -- | -- | -- | -- | -- | -- | -- | -- | -- | X | X | X | X | -- | -- | -- | X | X | X | -- | -- | -- | -- |
| *Triportheus elongatus* | O, M, P | X | X | X | X | X | X | X | X | X | X | X | X | X | X | X | X | X | X | X | X | X | X | -- | -- | X | X | X | X | X | X |
| **Acestrorhynchidae** |  |  |  |  |  |  |  |  |  |  |  |  |  |  |  |  |  |  |  |  |  |  |  |  |  |  |  |  |  |  |  |
| *Acestrorhynchus falcatus* | C, R, P | X | X | -- | X | -- | -- | X | X | -- | -- | -- | -- | -- | X | -- | -- | -- | X | -- | -- | -- | -- | X | X | -- | -- | -- | -- | -- | -- |
| *Acestrorhynchus falcirostris* | C R, P | X | X | X | X | X | X | X | X | X | X | X | X | X | X | -- | X | X | X | X | X | -- | X | X | X | X | X | X | X | X | X |
| **Cynodontidae** |  |  |  |  |  |  |  |  |  |  |  |  |  |  |  |  |  |  |  |  |  |  |  |  |  |  |  |  |  |  |  |
| *Cynodon gibbus* | C, R | -- | X | -- | -- | -- | -- | -- | -- | -- | X | X | -- | -- | -- | -- | -- | -- | -- | -- | -- | -- | X | -- | -- | -- | -- | -- | X | X | -- |
| *Hydrolycus scomberoides* | C, M | X | X | X | X | -- | X | -- | X | -- | X | -- | X | X | X | -- | -- | -- | X | -- | -- | -- | X | -- | X | -- | -- | -- | X | -- | X |
| *Rhaphiodon vulpinus* | C, M, P | -- | X | X | X | X | -- | -- | X | X | X | X | X | X | X | X | X | -- | X | X | -- | X | X | -- | -- | -- | -- | X | X | X | X |
| **Erythrinidae** |  |  |  |  |  |  |  |  |  |  |  |  |  |  |  |  |  |  |  |  |  |  |  |  |  |  |  |  |  |  |  |
| *Hoplerythrinus unitaeniatus* | C, R, B | -- | -- | X | -- | X | X | X | -- | -- | -- | X | X | -- | -- | -- | -- | X | X | X | -- | -- | -- | X | -- | X | X | -- | X | -- | -- |
| *Hoplias malabaricus* | C, R, B | X | X | X | X | X | X | X | X | X | X | X | X | X | X | -- | X | X | X | X | X | X | -- | X | X | X | X | -- | X | -- | -- |
| **Ctenoluciidae** |  |  |  |  |  |  |  |  |  |  |  |  |  |  |  |  |  |  |  |  |  |  |  |  |  |  |  |  |  |  |  |
| *Boulengerella lucius* | C, R | -- | -- | -- | -- | -- | -- | -- | -- | -- | -- | -- | -- | -- | -- | -- | -- | -- | -- | -- | -- | -- | -- | X | X | -- | -- | -- | -- | -- | -- |
| *Boulengerella maculata* | R | -- | -- | -- | -- | -- | -- | -- | -- | X | -- | -- | -- | -- | -- | -- | -- | -- | -- | -- | -- | -- | -- | X | X | -- | -- | -- | -- | -- | X |
| **SILURIFORMES** |  |  |  |  |  |  |  |  |  |  |  |  |  |  |  |  |  |  |  |  |  |  |  |  |  |  |  |  |  |  |  |
| **Cetopsidae** |  |  |  |  |  |  |  |  |  |  |  |  |  |  |  |  |  |  |  |  |  |  |  |  |  |  |  |  |  |  |  |
| *Cetopsis coecutiens* | R | -- | -- | -- | -- | -- | -- | -- | -- | -- | -- | -- | -- | -- | -- | -- | X | -- | -- | -- | -- | -- | X | -- | -- | -- | -- | -- | X | -- | -- |
| **Callichthyidae** |  |  |  |  |  |  |  |  |  |  |  |  |  |  |  |  |  |  |  |  |  |  |  |  |  |  |  |  |  |  |  |
| *Dianema longibarbis* | R | -- | -- | -- | -- | -- | -- | X | X | -- | -- | -- | X | -- | X | -- | -- | -- | -- | -- | -- | -- | -- | -- | -- | X | -- | -- | -- | -- | -- |
| *Dianema urostriatum* | R | -- | -- | -- | -- | -- | -- | X | X | -- | -- | X | X | -- | -- | -- | -- | -- | -- | -- | -- | -- | -- | -- | -- | -- | -- | -- | -- | -- | -- |
| *Hoplosternum littorale* | R, BT | X | -- | X | X | X | -- | X | X | -- | -- | X | X | X | X | X | X | X | X | X | -- | X | X | X | -- | X | X | X | -- | -- | -- |
| *Megalechis thoracata* | R | -- | -- | -- | -- | -- | -- | X | X | -- | -- | -- | -- | -- | -- | X | -- | -- | -- | X | -- | X | -- | X | -- | -- | -- | -- | -- | -- | -- |
| **Loricariidae** |  |  |  |  |  |  |  |  |  |  |  |  |  |  |  |  |  |  |  |  |  |  |  |  |  |  |  |  |  |  |  |
| *Ancistrus dolichopterus* | R | -- | -- | -- | -- | -- | -- | -- | -- | -- | -- | -- | -- | -- | -- | -- | -- | -- | -- | -- | -- | X | -- | -- | -- | -- | -- | -- | -- | -- | -- |
| *Ancistrus hoplogenys* | R | -- | -- | -- | -- | -- | -- | -- | X | -- | -- | -- | -- | -- | -- | -- | -- | -- | -- | X | -- | -- | -- | -- | -- | -- | -- | -- | -- | -- | -- |
| *Dekeyseria amazonica* | R | -- | -- | -- | -- | X | -- | -- | -- | X | -- | -- | X | -- | -- | X | X | -- | X | X | -- | X | X | -- | -- | -- | -- | X | X | X | -- |
| *Dekeyseria scaphirhyncha* | R | -- | X | -- | X | -- | -- | -- | -- | -- | -- | -- | -- | -- | -- | -- | -- | -- | -- | -- | -- | -- | -- | -- | -- | -- | -- | -- | -- | -- | -- |
| *Loricaria cataphracta* | R | -- | X | -- | -- | -- | X | -- | -- | -- | -- | -- | -- | -- | -- | -- | -- | X | X | X | X | -- | -- | -- | -- | X | X | -- | -- | -- | -- |
| *Loricaria lentiginosa* | R | -- | -- | -- | -- | -- | -- | -- | -- | -- | X | -- | -- | -- | -- | -- | -- | -- | -- | -- | -- | -- | -- | -- | -- | -- | -- | X | -- | -- | -- |
| *Loricariichthys maculatus* | R | -- | -- | -- | -- | -- | X | -- | -- | -- | -- | -- | -- | X | -- | -- | -- | -- | X | -- | X | -- | -- | X | X | -- | -- | X | -- | -- | -- |
| *Loricariichthys nudirostris* | R | -- | -- | -- | -- | -- | -- | -- | -- | X | -- | -- | -- | -- | X | -- | X | X | X | -- | X | -- | -- | X | X | X | X | -- | X | -- | X |
| *Hypoptopoma gulare* | R | -- | -- | X | -- | X | -- | -- | -- | -- | -- | X | -- | -- | -- | -- | X | -- | X | X | -- | -- | -- | -- | -- | X | -- | -- | -- | -- | X |
| *Hypoptopoma incognitum* | R | -- | -- | -- | -- | -- | -- | -- | -- | -- | -- | -- | -- | -- | -- | X | -- | -- | -- | -- | -- | -- | -- | -- | -- | -- | -- | -- | -- | -- | -- |
| *Hypostomus plecostomus* | R | -- | -- | X | -- | -- | -- | -- | -- | -- | -- | -- | -- | X | -- | X | -- | X | -- | -- | -- | -- | -- | -- | -- | -- | -- | -- | -- | -- | -- |
| *Pterygoplichthys pardalis* | R, BT | X | X | X | X | X | X | X | X | -- | -- | X | X | X | X | X | -- | X | X | X | X | -- | X | -- | -- | X | X | X | X | X | -- |
| *Squaliforma emarginata* | R, BT | X | X | X | X | -- | X | -- | -- | X | -- | -- | -- | -- | X | X | -- | -- | X | X | -- | -- | X | X | -- | X | -- | -- | -- | -- | -- |
| *Sturisoma lyra* | R | -- | -- | -- | -- | -- | -- | -- | -- | -- | -- | -- | -- | -- | -- | X | X | -- | X | -- | -- | -- | -- | X | -- | -- | -- | -- | -- | -- | -- |
| **Pimelodidae** |  |  |  |  |  |  |  |  |  |  |  |  |  |  |  |  |  |  |  |  |  |  |  |  |  |  |  |  |  |  |  |
| *Brachyplatistoma juruense* | C, M | -- | -- | -- | -- | -- | -- | -- | -- | -- | -- | -- | -- | -- | -- | -- | X | -- | -- | -- | -- | -- | -- | -- | -- | -- | -- | -- | -- | -- | -- |
| *Calophysus macropterus* | O, M, BT | -- | -- | -- | -- | -- | -- | -- | X | X | -- | -- | X | -- | X | X | X | -- | -- | X | -- | X | X | -- | -- | -- | -- | X | X | -- | X |
| *Hemisorubim platyrhynchos* | C, M, BT | -- | X | -- | -- | -- | -- | -- | -- | -- | -- | -- | -- | -- | X | -- | -- | -- | X | -- | -- | -- | -- | -- | -- | -- | -- | -- | X | -- | -- |
| *Hypophthalmus edentatus* | M, P | X | X | -- | -- | -- | X | -- | X | -- | -- | X | X | X | X | X | X | X | X | X | -- | X | X | X | -- | X | -- | X | X | X | X |
| *Hypophthalmus fimbriatus* | M | -- | -- | -- | -- | -- | -- | -- | -- | -- | X | -- | -- | -- | -- | -- | -- | -- | -- | -- | -- | -- | X | -- | -- | -- | -- | X | X | X | X |
| *Hypophthalmus marginatus* | M, P | X | -- | -- | X | -- | -- | -- | -- | X | X | X | X | -- | X | X | X | -- | X | -- | X | X | X | X | X | -- | -- | X | X | X | X |
| *Leiarius marmoratus* | M | -- | -- | -- | -- | -- | -- | -- | -- | -- | -- | -- | -- | -- | -- | -- | -- | -- | -- | X | -- | -- | -- | -- | -- | -- | -- | -- | -- | -- | -- |
| *Pharactocephalus hemioliopterus* | M, BT | -- | -- | -- | -- | -- | X | -- | -- | -- | -- | -- | -- | -- | -- | -- | -- | -- | -- | -- | -- | -- | -- | -- | -- | -- | -- | -- | -- | -- | -- |
| *Pimelodina flavipinnis* | C, M, BT | -- | X | -- | X | X | -- | -- | -- | -- | -- | -- | X | -- | -- | -- | -- | -- | X | -- | -- | -- | -- | -- | -- | -- | -- | -- | -- | -- | -- |
| *Pimelodus blochii* | O, R, BT | -- | X | X | X | X | X | X | X | X | X | X | X | X | X | X | X | X | X | X | X | X | X | X | X | X | X | X | X | X | X |
| *Pininampus pirinampu* | C, M, BT | -- | X | X | X | -- | -- | -- | -- | -- | -- | -- | X | -- | X | X | -- | X | X | -- | -- | X | X | -- | -- | -- | -- | -- | X | -- | X |
| *Platynematichthys notatus* |  | -- | -- | -- | -- | -- | -- | -- | -- | -- | -- | -- | -- | -- | -- | -- | X | X | -- | -- | -- | -- | -- | -- | -- | -- | -- | -- | -- | -- | -- |
| *Pseudoplatystoma fasciatum* | C, M, BT | -- | -- | X | -- | -- | X | -- | X | -- | -- | X | -- | X | X | -- | -- | X | X | -- | -- | -- | X | -- | X | X | -- | -- | X | -- | -- |
| *Pseudoplatystoma tigrinum* | C, M, BT | -- | X | -- | X | -- | X | -- | -- | -- | -- | -- | -- | X | X | -- | X | -- | X | X | X | -- | X | X | X | -- | -- | -- | X | -- | X |
| *Sorubim lima* | C, M, BT | -- | -- | -- | X | -- | X | X | -- | X | X | X | X | X | X | X | X | -- | -- | -- | -- | X | X | -- | -- | -- | -- | X | X | X | X |
| **Doradidae** |  |  |  |  |  |  |  |  |  |  |  |  |  |  |  |  |  |  |  |  |  |  |  |  |  |  |  |  |  |  |  |
| *Agamyxis pectinifrons* | R | -- | -- | -- | -- | -- | -- | -- | -- | -- | -- | -- | -- | -- | -- | -- | -- | -- | -- | -- | -- | -- | X | -- | -- | -- | -- | -- | -- | -- | -- |
| *Anadoras grypus* | R | -- | -- | -- | -- | -- | -- | X | X | -- | -- | -- | -- | X | -- | -- | -- | -- | -- | -- | -- | X | X | -- | -- | -- | -- | X | -- | -- | -- |
| *Astrodoras asterifrons* | R | -- | -- | -- | -- | -- | -- | -- | -- | -- | -- | X | -- | -- | -- | X | -- | -- | -- | -- | -- | X | -- | -- | -- | -- | -- | -- | -- | -- | -- |
| *Hemidoras stenopeltis* | R | -- | -- | -- | -- | -- | -- | -- | -- | -- | X | X | -- | -- | X | -- | -- | -- | -- | -- | -- | -- | X | -- | -- | -- | -- | X | -- | -- | -- |
| *Lithodoras dorsalis* | M | -- | -- | -- | -- | -- | -- | -- | -- | -- | -- | -- | -- | -- | -- | -- | -- | -- | -- | -- | -- | -- | -- | -- | -- | -- | -- | -- | X | -- | -- |
| *Megalodoras uranoscopus* | R | -- | -- | -- | -- | -- | -- | -- | -- | -- | -- | -- | -- | X | -- | -- | X | -- | -- | -- | -- | -- | X | -- | -- | -- | -- | -- | -- | -- | -- |
| *Nemadoras elongatus* | R | -- | -- | -- | -- | -- | -- | -- | -- | -- | -- | X | -- | -- | -- | -- | -- | -- | -- | -- | -- | -- | X | -- | -- | -- | -- | -- | -- | -- | -- |
| *Nemadoras hemipeltis* | R | -- | -- | -- | -- | -- | -- | -- | -- | X | -- | -- | -- | -- | -- | -- | -- | -- | -- | -- | -- | -- | -- | -- | -- | -- | -- | -- | -- | -- | -- |
| *Nemadoras humeralis* | R | -- | -- | -- | -- | -- | -- | X | -- | X | -- | -- | -- | -- | -- | -- | -- | -- | -- | -- | -- | -- | -- | -- | -- | -- | -- | -- | -- | -- | -- |
| *Ossancora punctatus* | R | -- | -- | -- | -- | -- | -- | X | -- | -- | -- | X | -- | -- | X | X | -- | X | -- | -- | -- | X | X | -- | -- | -- | -- | X | X | X | X |
| *Oxidoras niger* | O, R, BT | -- | X | X | X | -- | X | X | X | -- | -- | X | -- | X | X | X | X | X | X | X | X | -- | X | -- | -- | -- | -- | X | X | X | X |
| *Pterodoras granulosus* | H, M | -- | -- | -- | -- | -- | -- | -- | -- | -- | -- | -- | -- | X | -- | X | -- | -- | -- | -- | -- | X | X | -- | -- | -- | -- | X | X | -- | -- |
| *Scorpiodoras heckelii* | R | -- | -- | -- | -- | -- | -- | -- | -- | -- | -- | X | -- | -- | -- | -- | -- | -- | -- | -- | -- | -- | -- | -- | -- | -- | -- | -- | -- | -- | -- |
| *Trachydoras steindachneri* | R | -- | -- | -- | -- | -- | -- | -- | -- | -- | -- | -- | -- | -- | -- | -- | -- | X | -- | -- | -- | -- | -- | -- | -- | -- | -- | -- | -- | -- | -- |
| **Auchenipteridae** |  |  |  |  |  |  |  |  |  |  |  |  |  |  |  |  |  |  |  |  |  |  |  |  |  |  |  |  |  |  |  |
| *Ageneiosus atronasus* | R | -- | -- | -- | -- | -- | -- | -- | -- | -- | -- | -- | -- | X | -- | -- | -- | -- | -- | -- | -- | -- | X | -- | -- | -- | -- | -- | X | -- | -- |
| *Ageneiosus brevis* | R | -- | -- | -- | -- | -- | -- | -- | -- | X | X | X | X | X | X | X | -- | -- | -- | -- | -- | -- | -- | -- | -- | -- | -- | X | X | X | X |
| *Ageneiosus inermis* | C, R | -- | X | -- | X | -- | -- | -- | -- | X | -- | X | X | X | X | X | X | X | X | X | -- | -- | X | -- | -- | -- | -- | X | X | -- | X |
| *Ageneiosus piperatus* | R | -- | -- | -- | -- | -- | -- | -- | -- | X | -- | -- | -- | -- | -- | -- | -- | -- | -- | -- | -- | -- | -- | -- | -- | -- | -- | X | -- | -- | X |
| *Ageneiosus vittatus* | R | -- | -- | -- | -- | -- | -- | X | X | X | X | X | X | X | X | -- | -- | -- | -- | -- | -- | -- | -- | -- | -- | -- | -- | -- | -- | X | -- |
| *Ageneiosus ucayalensis* | C, R | -- | -- | -- | -- | -- | -- | -- | -- | -- | -- | -- | -- | -- | X | X | X | -- | -- | -- | -- | X | X | -- | -- | -- | -- | -- | X | X | X |
| *Auchenipterichthys thoracatus* | R | -- | -- | -- | -- | -- | -- | -- | -- | -- | -- | -- | -- | -- | -- | X | -- | X | -- | -- | -- | X | X | -- | -- | -- | -- | -- | -- | X | X |
| *Auchenipterus britskii* | R | -- | -- | -- | -- | -- | -- | X | -- | -- | -- | X | -- | -- | -- | -- | -- | -- | -- | -- | -- | X | X | -- | -- | -- | -- | -- | -- | X | X |
| *Auchenipterus dentatus* | C, R | X | X | -- | -- | -- | -- | X | -- | -- | -- | X | -- | -- | X | X | X | -- | X | X | -- | -- | X | -- | -- | -- | -- | -- | X | X | X |
| *Auchenipterus nuchalis* | C, R | X | X | -- | -- | X | -- | -- | -- | X | X | X | X | X | X | X | X | X | X | X | -- | X | X | -- | -- | -- | -- | X | X | X | X |
| *Centromochlus heckelii* | R | -- | -- | -- | -- | -- | -- | -- | -- | X | X | -- | -- | -- | -- | -- | X | -- | -- | -- | -- | -- | X | -- | -- | -- | -- | -- | -- | X | X |
| *Epapterus dispilurus* | C, R | -- | -- | -- | -- | -- | -- | -- | -- | -- | -- | -- | -- | -- | -- | X | -- | -- | -- | -- | -- | -- | -- | -- | -- | -- | -- | -- | -- | -- | X |
| *Trachelyopterichthys taeniatus* | R | -- | -- | -- | -- | -- | -- | -- | -- | -- | X | -- | -- | -- | -- | -- | -- | -- | -- | -- | -- | -- | -- | -- | -- | -- | -- | -- | -- | -- | X |
| *Trachelyopterus galeatus* | O, R, BT | -- | -- | -- | -- | -- | -- | X | X | X | X | X | X | X | X | X | X | -- | X | X | -- | X | X | -- | -- | X | -- | X | X | X | X |
| *Trachycorystes porosus* | R | -- | -- | -- | -- | -- | -- | -- | -- | -- | -- | X | -- | -- | X | X | -- | -- | -- | -- | -- | X | X | -- | -- | -- | -- | -- | -- | -- | -- |
| *Tatia intermedia* | C, R | -- | -- | -- | -- | -- | -- | -- | -- | X | X | -- | -- | -- | -- | -- | -- | -- | -- | -- | -- | -- | -- | -- | -- | -- | -- | -- | X | -- | -- |
| **GYMNOTIFORMES** |  |  |  |  |  |  |  |  |  |  |  |  |  |  |  |  |  |  |  |  |  |  |  |  |  |  |  |  |  |  |  |
| **Gymnotidae** |  |  |  |  |  |  |  |  |  |  |  |  |  |  |  |  |  |  |  |  |  |  |  |  |  |  |  |  |  |  |  |
| *Electrophorus electricus* | R | -- | -- | -- | -- | -- | -- | -- | -- | -- | -- | -- | -- | -- | -- | -- | -- | -- | -- | X | -- | -- | -- | -- | -- | -- | X | -- | -- | -- | -- |
| *Gymnotus carapo* | C, R | -- | X | -- | -- | -- | -- | X | X | -- | -- | X | -- | -- | -- | -- | -- | -- | -- | -- | -- | -- | -- | -- | -- | -- | -- | -- | -- | -- | -- |
| **Sternopygidae** |  |  |  |  |  |  |  |  |  |  |  |  |  |  |  |  |  |  |  |  |  |  |  |  |  |  |  |  |  |  |  |
| *Eigenmannina macrops* | R | -- | -- | -- | -- | -- | -- | -- | -- | -- | -- | -- | -- | -- | -- | -- | -- | -- | X | -- | -- | -- | -- | -- | -- | -- | -- | -- | -- | -- | -- |
| *Sternopygus macrurus* | C, R | -- | X | -- | -- | -- | -- | -- | -- | -- | -- | X | X | -- | -- | -- | X | -- | -- | -- | -- | -- | -- | -- | -- | -- | -- | -- | -- | -- | -- |
| **Rhamphichthyidae** |  |  |  |  |  |  |  |  |  |  |  |  |  |  |  |  |  |  |  |  |  |  |  |  |  |  |  |  |  |  |  |
| *Rhamphichthys rostratus* | C, R | -- | -- | -- | -- | -- | -- | -- | X | -- | -- | -- | -- | -- | -- | -- | -- | -- | -- | -- | -- | -- | -- | -- | -- | -- | -- | -- | -- | -- | X |
| **Apteronotidae** |  |  |  |  |  |  |  |  |  |  |  |  |  |  |  |  |  |  |  |  |  |  |  |  |  |  |  |  |  |  |  |
| *Parapteronotus hasemani* | R | -- | -- | -- | -- | -- | -- | -- | -- | -- | -- | -- | X | X | -- | -- | -- | -- | -- | -- | -- | -- | X | -- | -- | -- | -- | -- | -- | -- | -- |
| **SYNBRANCHIFORMES** |  |  |  |  |  |  |  |  |  |  |  |  |  |  |  |  |  |  |  |  |  |  |  |  |  |  |  |  |  |  |  |
| **Synbranchidae** |  |  |  |  |  |  |  |  |  |  |  |  |  |  |  |  |  |  |  |  |  |  |  |  |  |  |  |  |  |  |  |
| *Synbranchus marmoratus* | R | -- | -- | -- | -- | -- | -- | -- | -- | -- | -- | -- | -- | -- | -- | -- | -- | -- | -- | -- | -- | X | -- | -- | -- | -- | -- | -- | -- | -- | -- |
| **PERCIFORMES** |  |  |  |  |  |  |  |  |  |  |  |  |  |  |  |  |  |  |  |  |  |  |  |  |  |  |  |  |  |  |  |
| **Sciaenidae** |  |  |  |  |  |  |  |  |  |  |  |  |  |  |  |  |  |  |  |  |  |  |  |  |  |  |  |  |  |  |  |
| *Plagioscion squamosissimus* | C, R, B | -- | -- | -- | -- | X | X | -- | -- | X | X | -- | X | -- | X | -- | X | -- | X | X | -- | -- | X | -- | -- | -- | -- | -- | X | X | X |
| **Cichlidae** |  |  |  |  |  |  |  |  |  |  |  |  |  |  |  |  |  |  |  |  |  |  |  |  |  |  |  |  |  |  |  |
| *Acarichthys heckelii* | H, R, B | -- | X | -- | -- | -- | X | -- | -- | -- | X | -- | -- | -- | -- | -- | -- | -- | X | X | X | -- | -- | X | X | X | X | X | X | -- | -- |
| *Acaronia nassa* | C, R | -- | -- | -- | -- | X | -- | -- | -- | X | X | -- | -- | -- | -- | -- | -- | -- | -- | -- | -- | -- | -- | -- | -- | X | X | -- | -- | -- | -- |
| *Astronotus crassipinnis* | O, R, B | X | X | -- | X | X | X | X | X | X | X | -- | -- | -- | X | -- | -- | X | X | -- | -- | -- | -- | -- | -- | X | X | X | -- | -- | -- |
| *Astronotus ocellatus* | C, R, B | X | X | X | X | X | X | -- | -- | -- | -- | X | -- | -- | -- | -- | -- | X | -- | X | X | -- | -- | -- | X | X | X | X | -- | -- | -- |
| *Biotodoma cupido* | R | -- | -- | -- | -- | -- | -- | -- | -- | -- | -- | -- | -- | -- | -- | -- | -- | -- | -- | X | -- | -- | -- | -- | -- | -- | -- | -- | -- | -- | -- |
| *Chaetobranchopsis orbiculares* | R, B | X | X | -- | -- | X | X | X | X | -- | -- | -- | -- | -- | -- | -- | -- | -- | -- | -- | -- | X | X | -- | X | X | X | -- | -- | -- | -- |
| *Chaetobranchus flavescens* | R, B | X | -- | X | X | -- | X | X | X | X | X | -- | -- | -- | -- | -- | -- | -- | X | X | X | -- | -- | X | X | X | X | -- | -- | -- | -- |
| *Chaetobranchus semifasciatus* | R, B | -- | -- | X | X | -- | X | -- | -- | -- | -- | -- | -- | -- | -- | -- | -- | -- | -- | X | X | -- | -- | -- | -- | -- | X | -- | -- | -- | -- |
| *Cichla monoculus* | C, R, P | X | X | X | X | X | X | X | X | X | X | X | X | X | X | -- | X | X | X | X | X | X | X | -- | X | X | X | X | X | X | X |
| *Cichlasoma amazonarum* | O, R, B | X | X | X | X | X | X | -- | X | -- | -- | -- | -- | -- | -- | -- | -- | X | -- | X | X | -- | -- | -- | -- | -- | X | X | -- | -- | -- |
| *Crenicichla cincta* | R | X | -- | -- | -- | -- | -- | -- | -- | X | X | -- | -- | -- | -- | -- | X | -- | -- | -- | -- | -- | X | -- | -- | -- | -- | -- | -- | -- | -- |
| *Crenicichla macrophthalma* | R | -- | -- | X | -- | -- | -- | -- | -- | -- | -- | -- | -- | -- | -- | -- | -- | -- | -- | -- | -- | -- | -- | -- | -- | -- | -- | -- | -- | -- | -- |
| *Crenicichla reticulata* | R | -- | X | X | X | -- | X | -- | -- | X | -- | -- | -- | -- | -- | -- | -- | -- | X | -- | -- | -- | -- | -- | -- | -- | -- | -- | -- | -- | -- |
| *Crenicichla strigata* | R | -- | -- | -- | -- | -- | -- | -- | -- | -- | -- | -- | -- | -- | -- | -- | -- | -- | -- | -- | -- | -- | -- | -- | -- | -- | -- | -- | -- | X | -- |
| *Geophagus altifrons* | R, B | -- | -- | -- | -- | X | X | -- | X | X | X | X | -- | -- | X | -- | -- | -- | -- | X | X | -- | -- | -- | X | X | X | X | -- | -- | -- |
| *Heros severus* | H, R, B | X | X | X | X | X | X | -- | -- | X | X | X | -- | -- | X | -- | -- | -- | X | X | X | -- | X | X | X | X | X | X | -- | -- | X |
| *Hypselecara temporalis* | C, R | -- | -- | -- | -- | -- | -- | -- | -- | -- | -- | -- | -- | -- | -- | -- | -- | -- | -- | X | -- | -- | -- | -- | X | -- | -- | -- | -- | -- | -- |
| *Mesonauta festivus* | R | X | -- | X | -- | X | X | X | X | X | X | X | X | -- | X | X | X | -- | X | X | X | X | X | X | X | X | X | X | X | X | X |
| *Pterophyllum scalare* | O, R, P | -- | -- | -- | -- | -- | X | -- | -- | -- | -- | -- | -- | -- | -- | X | -- | X | -- | X | X | -- | -- | X | X | X | X | -- | X | -- | -- |
| *Satanoperca acuticeps* | O, R, B | -- | -- | -- | -- | -- | X | -- | -- | X | X | X | -- | X | -- | -- | -- | -- | -- | X | X | -- | X | X | X | X | -- | -- | -- | -- | -- |
| *Satanoperca jurupari* | O, R, B | -- | -- | X | X | -- | X | -- | -- | X | X | X | -- | -- | -- | -- | -- | -- | X | X | X | X | -- | X | X | X | X | -- | -- | -- | -- |
| *Symphysodon aequifasciatus* | C, R | -- | -- | -- | -- | -- | -- | -- | -- | -- | X | -- | -- | -- | -- | -- | -- | -- | -- | -- | -- | -- | -- | -- | -- | -- | -- | -- | -- | -- | -- |
| *Uaru amphiacanthoides* | H, R, P | -- | -- | -- | -- | -- | -- | -- | -- | X | X | -- | -- | -- | X | -- | -- | -- | -- | -- | -- | -- | -- | X | -- | -- | X | -- | -- | -- | -- |
| **Tetraodontiformes** |  |  |  |  |  |  |  |  |  |  |  |  |  |  |  |  |  |  |  |  |  |  |  |  |  |  |  |  |  |  |  |
| **Tetraodontidae** |  |  |  |  |  |  |  |  |  |  |  |  |  |  |  |  |  |  |  |  |  |  |  |  |  |  |  |  |  |  |  |
| *Colomesus asellus* | C, R | -- | -- | -- | -- | -- | -- | -- | -- | X | -- | -- | -- | -- | -- | -- | -- | -- | -- | -- | -- | -- | -- | -- | -- | -- | -- | -- | -- | -- | -- |

H = How water; L = Low water

Type: B = Bentonic; BT = Bentopelagic; P = pelagic; H = Herbivorous; C = carnivorous; O = omnivorous; M = migrator; R = resident
